# Effect of radiation therapy on lymph node fluorescence in head and neck squamous cell carcinoma after intravenous injection of indocyanine green: a prospective evaluation

**DOI:** 10.1186/s13550-024-01106-5

**Published:** 2024-05-16

**Authors:** Antoine Digonnet, Sophie Vankerkhove, Michel Moreau, Cécile Dekeyser, Marie Quiriny, Esther Willemse, Nicolas de saint Aubain, Matteo Cappello, Vincent Donckier, Pierre Bourgeois

**Affiliations:** 1 Department of Head and Neck Surgery, Jules Bordet Institute, Université Libre de Bruxelles, 93 Rue Meylemeerch, Brussels, 1070 Belgium; 2 Department of Surgical Oncology, Jules Bordet Institute, Université Libre de Bruxelles, Brussels, Belgium; 3 Department of Biostatistics, Jules Bordet institute, Université Libre de Bruxelles, Brussels, Belgium; 4 Department of Pathology, Jules Bordet institute, Université Libre de Bruxelles, Brussels, Belgium; 5 Department of Thoracic Surgery, Academic Erasmus Hopsital, Université Libre de Bruxelles, Brussels, Belgium; 6 Department of Surgery, Jules Bordet institute, Université Libre de Bruxelles, Brussels, Belgium; 7 Department of Nuclear Medicine, Academic Erasmus Hopsital, Université Libre de Bruxelles, Brussels, Belgium

**Keywords:** ICG, Head & neck squamous cell carcinoma, Radiation therapy

## Abstract

**Background:**

Indocyanine green (ICG)-guided surgery has proven effective in the identification of neoplastic tissues. The effect of radiation therapy (RT) on lymph node fluorescence after intravenous injection of ICG has not been addressed yet. The objective of this study was to evaluate the influence of RT on node fluorescence during neck dissection in head and neck squamous cell carcinoma (HNSCC).

**Results:**

Twenty-four patients with planned neck dissection for HNSCC were prospectively enrolled. Eleven were included without previous radiation therapy and 13 after RT. ICG was intravenously administered in the operating room. The resected specimen was analyzed by the pathology department to determine the status of each resected lymph node (invaded or not). The fluorescence of each resected node was measured in arbitrary units (AU) on paraffin blocs. The surface area (mm^2^) of all metastatic nodes and of the invaded component were measured. The values of these surface areas were correlated to fluorescence values. A total of 707 nodes were harvested, the mean fluorescence of irradiated nodes (*n* = 253) was 9.2 AU and of non-irradiated nodes (*n* = 454) was 9.6 AU (*p* = 0.63). Fifty nodes were invaded, with a mean fluorescence of 22 AU. The mean fluorescence values in the invaded irradiated nodes (*n* = 20) and the invaded non-irradiated nodes (*n* = 30) were 19 AU and 28 AU (*p* = 0.23), respectively. The surface area of metastatic nodes and of the invaded component were correlated to fluorescence values even after previous RT (*p* = 0.02).

**Conclusion:**

No differences were observed between the fluorescence of irradiated and non-irradiated lymph nodes, including invaded nodes. ICG-guided surgery can be performed after failed RT.

**Trial registration:**

EudraCT ref. 2013-004498-29, registered 29 November 2013. https://www.clinicaltrialsregister.eu/ctr-search/search?query=2013-004498-29

## Background

Fluorescence imaging (FI) after intravenous injection of indocyanine green (ICG) has been demonstrated to be an effective technique for in vivo identification of tumour tissues and associated metastatic lymph nodes in head and neck cancers [1]. Radiation therapy (RT) is a mainstay of care in locally advanced head and neck squamous cell carcinoma (HNSCC) but neck dissections must often be performed after failed radiation therapy [2].

In these cases, the feasibility of FI after ICG injection has not been verified. In fact, ionizing radiation induces damage, not only in rapidly proliferating cancer cells, but also in normal tissue in the radiation field [3]. An important late effect of RT that contributes to patient morbidity is radiation-induced fibrosis (RIF). RIF usually occurs 4–12 months after treatment, progresses over several years, and manifests as skin induration and thickening, muscle shortening and atrophy, and limited joint mobility [4]. RIF is characterized by the aberrant growth of myofibroblasts that leads to an excess of collagen that reduces vascularity over time [5]. This decreased vascularization makes fibrotic areas susceptible to physical trauma and gradual ischemia, which may lead to necrosis [6].

On this basis, we hypothesized that RT therapy may change the optical properties of tissue, hindering access of molecules to the tumour, and impacting lymph node (LN) fluorescence after intravenous (IV) injection of ICG. The aim of this prospective study was to evaluate the influence of RT on LN fluorescence after intravenous ICG injection in a homogeneous series of patients with head and neck squamous cell carcinoma (HNSCC).

## Methods

This clinical trial was approved by the medical ethics committee (CE:2178) of the Institute Jules Bordet, Université Libre de Bruxelles.

Patients with a history of renal failure and coronaropathy were excluded. The decision to perform neck dissection with or without a resection of the primary tumour was made during multidisciplinary oncologic consultations. Twenty-four patients with HNSCC from a single institution were prospectively enrolled, including 11 without previous RT and 13 with RT before the surgery. Patients scheduled for neck dissection who had less than 50 Gy exposure to their lymph nodes were excluded. The time period, in months, between RT and surgery was recorded.

Free ICG (0.25 mg/kg; Verdeye ® Pulsion Medical Systems, Belgium) was injected through thecephalic vein at the induction time of anesthesia. Patients underwent LN dissection with or without primary resection as previously planned. The resected specimen was sent to the department of pathology where microscopic analysis was performed for each LN to determine its status (invaded or not). The analysis of LN fluorescence was blinded to node status. The fluorescence of each resected LN was measured in arbitrary units (AU) on paraffin blocs. Arbitrary unit was defined as the ratio between the emission light intensity in the 420–600 nm range and the excitation light intensity between 300 and 420 nm. The ration was calculated by the software IC-calc 2.0.

The surface area (mm^2^) of all metastatic nodes was measured according to each node’s large and small diameter. Using region of interest (ROI) software (IC-calc 2.0), the surface area of the invaded component of each metastatic node was also measured. Finally, the surface area of the metastatic node and the surface area of the invaded component of metastatic LN were correlated to fluorescence values.

### Statistical analysis

As the unit of analysis is the LN and not the patient, the data are not independent. In order to consider the within-patient correlation structure, a mixed linear model was used to compare the mean fluorescence values between irradiated and non-irradiated patients and a generalized estimating equations (GEE) model was used to analyze the associations between the surface area of the LNs, the metastatic component of the LNs, and fluorescence values. In the GEE analysis, empirical instead of model-based standard errors were used since they are more robust for avoiding misspecification of the correlation structure. For both models, compound symmetry was used as a type of covariance matrix.

surface area between invaded irradiated node and invaded non-irradiated node were compared. Using Pearson’s correlation coefficient, we also evaluated the influence of the metastatic node surface to the value of fluorescence (*n* = 50). The same evaluation was performed in both subgroups, Irradiated nodes (*N* = 20) and non-irradiated nodes (*N* = 30).

For the analysis of the association between fluorescence and the surface area the metastatic component of the LNs, the P50 (among the positive LNs) was used to dichotomize the fluorescence. For the figures, a Pearson correlation analysis was used.

## Results

### Patient and tumour characteristics

Twenty-four patients were included, including 13 with preoperative RT on the primary tumour and draining LN field and 11 patients who underwent surgery without prior radiotherapy. There were 19 men and 7 women, with a mean age of 60 years old. The characteristics of the patients, tumours, and procedures performed are detailed in Table 1. In the irradiated patients, LN dissection was performed after a mean time of 31.4 months (min: 5; max 204). Twenty-one patients underwent LN dissection with resection of the primary tumour and 5 patients underwent LN dissection only for node recurrence after failed RT.

### Lymph node characteristics

A total of 707 LNs were harvested from 24 patients (Mean: 29.5 LN/patient, SD: 17.3), including 454 LNs in the 11 non-irradiated patients (Mean: 38.5, SD: 16.7) and 253 in the 13 irradiated patients (Mean: 19.4 SD: 9.8). Fifty LNs were invaded at pathological examination, including 30 in the non-irradiated patients and 20 in the irradiated patients.

**Table 1:** Patient characteristics, performed procedures, LN status, and previous RT

*N*° Patient	Age	Primary	C TNM	Resection of primary lesion	Neck dissection	No. of harvested LNs	No. of invaded LNs	Previous RT
1	66	Larynx	T4N0	Total Laryngectomy	II-IV left/right, VI	28	1	NO
2	55	Larynx	T4NO	Total Laryngectomy	II-IV left/right, VI	35	0	NO
3	63	Oral cavity	T2No	Glossectomy	I-III left/right	54	0	
4	47	Oral cavity	T4AN1	Mandibulectomy	I-III left	9	1	YES
5	68	Oropharynx	T2N1	Oropharyngectomy	I-III right	41	1	NO
6	72	Scalp	N2B	NO	parotidectomy-II-III	15	4	NO
7	60	Larynx	T3N2BMO	Total Laryngectomy	II-III left/right VI	45	2	NO
8	78	Larynx	T2N0MO	Total Laryngectomy	II-III left/right VI	21	0	YES
9	55	Piriform sinus	T4N2BMO	Total Laryngectomy	II-IV left/right, VI	42	3	NO
10	52	larynx	T2N2C	Total Laryngectomy	II-IV BILAT	28	3	YES
11	68	oropharynx	N3B	NO	II-IV-VA	3	2	YES
12	70	Piriform sinus /Oesophagus	T4AN2-TIS	Total Laryngectomy Oesophagectomy	II-IV-VI left	26	4	YES
13	61	Oropharynx	N3B	No	II-II-V right	5	1	YES
14	55	Piriform sinus	T4AN2C	Total Laryngectomy	II-IV left/right, VI	36	10	NO
15	56	Oropharynx	T1N2B	NO	II-IV	11	2	YES
16	50	Larynx	T3N1M0	Total Laryngectomy	II-IV left/right, VI	18	2	YES
17	60	Larynx	T2N1MO	Total Laryngectomy	II-IV left, VI	11	1	YES
18	56	Piriform sinus	T4A N2B	Total Laryngectomy	II-IV left/right, VI	70	4	NO
19	66	Larynx	T4AN2cMO	Total Laryngectomy	II-IV left/right, VI	52	4	NO
20	55	Piriform sinus	T2NOM0	Total Laryngectomy	II-IV right	13	0	YES
21	68	Larynx	T4NO	Total Laryngectomy	II-IV left/right, VI	52	2	YES
22	63	Larynx	T2NOM0	Total Laryngectomy	II-IV left/right	33	0	YES
23	64	Oral cavity	T2N0MO	Glossectomy	I-III left/right	53	1	NO
24	61	Oropharynx	N1	NO	I-III right	8	2	YES

LN: lymph node; radiation therapy;

### Ex-vivo lymph node fluorescence analysis

The mean fluorescence of the 707 resected LNs from the 24 patients was 9.5 AU. The mean LN fluorescence was 9.2 AU for LNs from irradiated patients and 9.6 AU for LNs from non-irradiated patients (*p* = 0.63). The mean fluorescence of the 50 invaded nodes was 22.6 AU (SD: 15.3), with a mean fluorescence of 28.1 AU (SD: 19.3) in the 20 irradiated nodes and 19.0 AU (SD: 11.0) in the 30 non-irradiated nodes. The difference in fluorescence between the irradiated and non-irradiated invaded nodes was not significant (*p* = 0.23). The mean surface area of the metastatic nodes was 204.0 mm² (P50 = 165). The mean surface in irradiated nodes and non-irradiated nodes was respectively 276,2 mm^2^(sd 133,1) and 155,9 mm^2^ (sd 166,7) *p* = 0.0095.

**Fig. 1:**
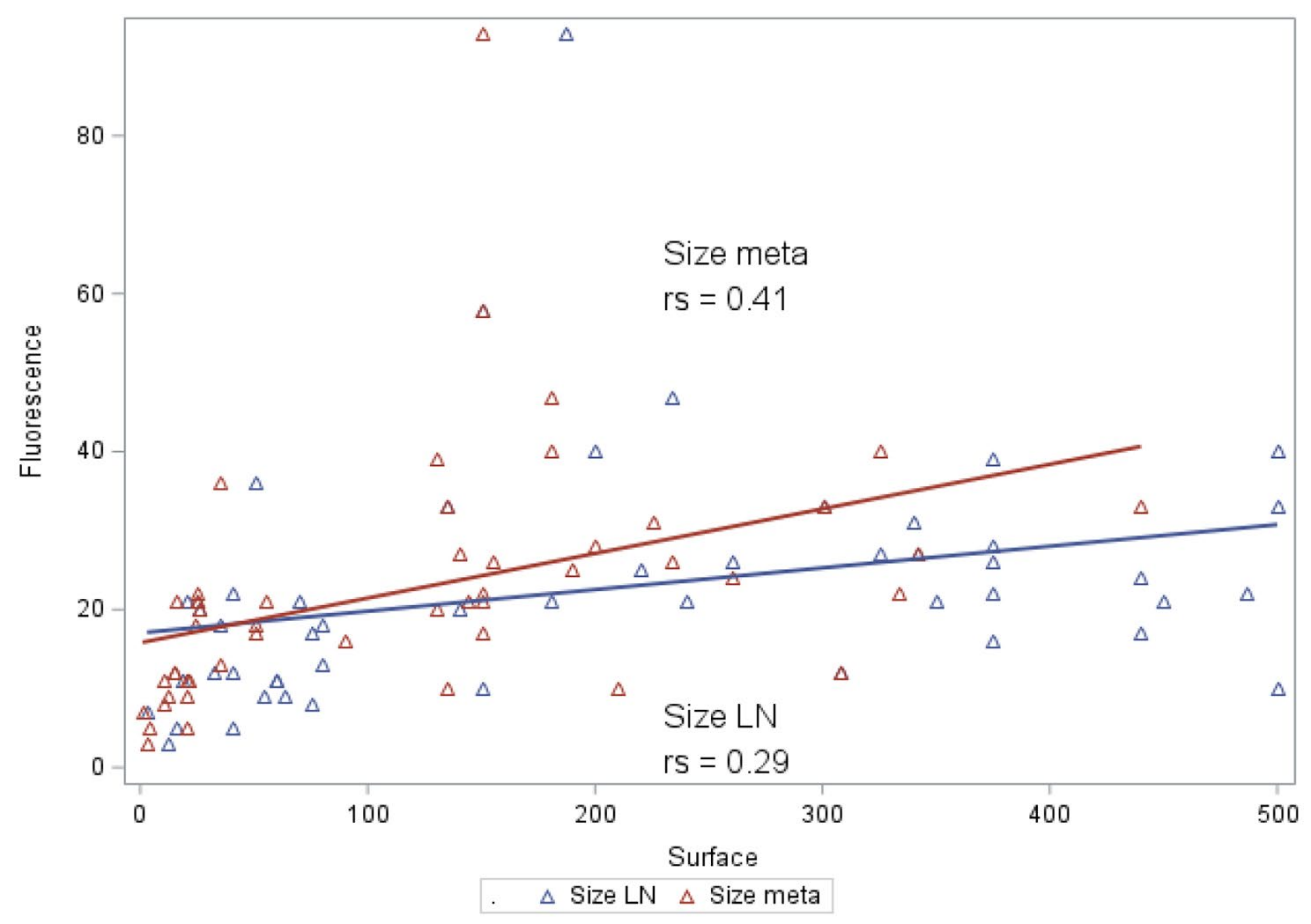
Correlation between the surface area of the metastatic nodes/ the metastatic component and the value of fluorescence

The mean surface area of the metastatic component was 121.1 mm² (P50 = 130).

correlation was observed between the surface area of the metastatic nodes (*N* = 50) and the value of fluorescence, Pearson’s correlation coefficient was 0.29 (*p* = 0,039) (Fig. 1).

Fluorescence was also correlated with surface of the nodes in non-irradiated patients (*N* = 30), Pearson’s correlation coefficient 0,56 (*p* = 0,0012).

Fluorescence was not correlated with surface of the nodes in irradiated patients (*N* = 20), Pearson’s correlation coefficient − 0,13 (*p* = 0,56) the surface area of the metastatic component (Fig. 1) was correlated with the value of fluorescence.

After adjustment for the presence of previous RT, the surface area of the metastatic nodes and the surface area of the metastatic component still correlated with the value of node fluorescence (Table 2). The image illustrates that only the invaded part of the metastatic node exhibits fluorescence.

**Table 2:** Correlation between surface area of invaded node, surface area of metastatic component, metastatic ratio, and node florescence value

	OR (95% CI)	*p*	Adjusted OR for RT (95%CI)	*p*
Surface area of the metastatic node				
≤ 165 mm²	1		1	
> 165 mm²	74.4 (3.3–35.5)	0.006	15.4 (2.9–80.3)	0.02
Surface area of the metastatic component of LN				
≤ 130 mm²	1		1	
> 130 mm²	12.1 (3.7–38.9)	0.007	10.03 (3.2–31.6)	0.02

CI: confidence interval; OR: odds ratio; RT: radiation therapy

**Figure:**
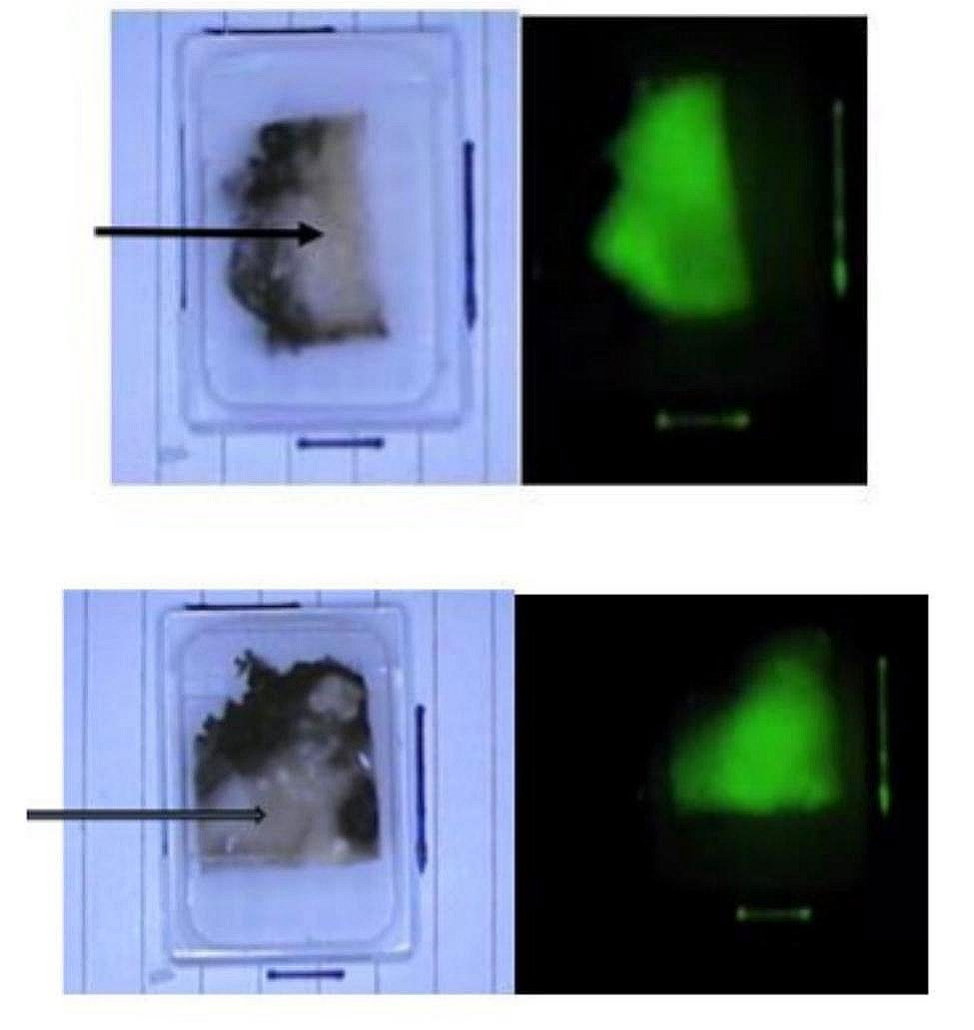
Image: Paraffin bloc, on the left-side picture with ambient light and on the right-side picture under near infra-red imaging. The arrow demonstrates the invaded part of the node

## Discussion

Recently, ICG-guided surgery has gained in popularity, exemplified by Xia et al. [7] who reported a sensitivity of 98.1% and specificity of 89.1% in the detection of metastatic nodes. In addition, a recent study [8] demonstrated the ability of ICG to identified neck metastases in a population of young adults and children with a sensitivity of 83%, a specificity of 88%, a positive predictive value (PPV) of 91%, and a negative predictive value (NPV) of 86%. Another recent study reported a sensitivity of 100% for pathologic nodes during neck dissection [9]. However, all of these studies were performed in malignancies that were not treated previously with radiation therapy.

Locally advanced HNSCC mainly requires multimodal treatment and RT, with or without chemotherapy, is often the first therapeutic option proposed to these patients [10, 11]. However, the aggressive nature of these cancers often leads to recurrence and, in this setting, surgery remains the only curative option [12]. As compared with first-line surgery, salvage interventions after RT are associated with higher rates of morbidity [13]. For neck dissection, in particular, RIF can impair visual and palpatory assessment, making it more difficult to distinguish between healthy, fibrotic, and neoplastic tissue.

In a previous study [14] in a heterogeneous population in terms of primary head and neck tumours and previous RT, we evaluated the feasibility of near infrared (NIR) fluorescent imaging after intravenous injection of ICG during neck dissection. We found that the presence of a fluorescent node was associated with a 14.1-fold risk of invasion regardless of the size of the node. In this feasibility study, fluorescence magnitude was correlated to node invasion and was able to distinguish between healthy and invading nodes after IV injection. However, we acknowledged that larger and more homogenous series were required to define the optimal role of NIR fluorescence in head and neck cancer and its potential routine utilization. Indeed, in this study, samples from one patient did not exhibit node fluorescence after IV injection of ICG. This patient underwent two sessions of radiotherapy and one surgery with node dissection before the last procedure with fluorescence imaging. For this reason, we decided to perform an objective measurement of a homogeneous population of patients with HNSCC with two comparable subgroups (with or without previous RT). However, more irradiated patients were included in the study since it has been established that the yield of lymph nodes is decreased in irradiated patients compared to non-irradiated patients [15].

To the best of our knowledge, the question of the influence of radiation therapy on node fluorescence in a population of patients with HNSCC has not been addressed yet. However, a recent study [16] assessed, for the first time, the influence of radiotherapy on the fluorescence of primary HNSCC after IV injection of ICG. Four patients with primary tumour recurrence after radiotherapy were included. Two patients had subjectively increased fluorescence compared to surrounding tissue. The last two patients had a tumour developed on a bed of lichen, one had moderately increased fluorescence compared to adjacent tissue and the last patient had no difference in fluorescence between the tumour and healthy tissue. The authors concluded that NIR fluorescence mapping in HNSCC patients previously subjected to radical radiotherapy clearly established the feasibility of using this technique to delineate tumours from lichen.

Based on these observations, it could be suspected that specific limitations of this fluorescence imaging technique could be encountered in patients who have been previously irradiated in the same region. Accordingly, the objective of the present work was to prospectively verify the feasibility of fluorescence imaging to detect LNs associated with HNSCC in patients who had undergone previous RT, as compared with those who did not receive RT before surgery.

Our main observation was that fluorescent LNs could be identified in patients who were previously irradiated, and regarding the total number of harvested nodes in the irradiated and non-irradiated populations, we did not observe a difference in fluorescence values (*p* = 0.63). Furthermore, we did not observe a significant difference between irradiated and non-irradiated metastatic nodes (*p* = 0.23). However, the average fluorescence of non-irradiated metastatic lymph nodes was 28.1 AU vs. 19AU in irradiated patients, suggesting a trend towards a decreased fluorescence in metastatic nodes in irradiated patients.

To better understand the factors influencing the value of fluorescence, we correlated the size of the metastatic node and the size of the metastatic component to the value of fluorescence. We found that the surface of the metastatic node and the metastatic component were correlated to the fluorescence value (*p* = 0.006 and *p* = 0.007, respectively), confirming our previous observations [14].

However, regarding the subgroup of irradiated patients, we did not find a correlation between the surface of the metastatic node and the fluorescence. This observation could be explained by necrotic component in irradiated nodes. In our serie, histopathological reports described necrotic component among nodes in 3 irradiated patients.

After adjustment for a previous RT, the correlation between the surface of a metastatic node and the size of the metastatic component remained significant (*p* = 0.02). This result suggests that the value of fluorescence is correlated to the amount of neoplastic tissue inside the invaded node. Therefore, the technique will probably be ineffective for the identification of micrometastases. However recent studies have shown in non-irradiated patients an in vivo sensibility of 100% in the identification of nodes metastasis and the identification of nodes metastasis outside the planned resection area [17, 18].

Thus, the main clinical application of the technic would probably be the identification of nodes metastasis outside the planned neck dissection area.

## Conclusion

This prospective study in a homogeneous population of patients with HNSCC indicates that fluorescence imaging after ICG intravenous injection is feasible in patients who have been previously irradiated. This technique could, therefore, represent a valuable tool for identifying, resecting, and analyzing LNs in this specific population where the detection of LNs and metastatic LNs could be particularly difficult according to classical methods.

## Data Availability

The datasets generated during and/or analysed during the current study are available from the corresponding author on reasonable request.

## Abbreviations

AU: Arbitrary units
FI: Fluorescence imaging
GEE: Generalized estimating equations
HNSCC: Head and neck squamous cell carcinoma
ICG: Indocyanine green
IV: Intravenous
LN: Lymph node .
RIF: Radiation-induced fibrosis
ROI: Region of interest
RT: Radiation therapy
